# Use of Pattern Recognition Analysis to Identify Underlying Relationships of Doxorubicin Derivatives Optimized for Breast Cancer Treatment

**DOI:** 10.5402/2011/585192

**Published:** 2011-06-30

**Authors:** Ronald Bartzatt

**Affiliations:** Chemistry Department, Durham Science Center, University of Nebraska at Omaha, 6001 Dodge Street, Omaha, NE 68182, USA

## Abstract

*Introduction*. Treatment of breast cancer includes surgery, drugs (hormone therapy and chemotherapy), and radiation. A discussion of eight drug constructs for the treatment of breast cancer, derived through application of in silico optimized molecular properties and substituent substitution, are analyzed using pattern recognition techniques. *Methods and Materials*. Determined properties of these eight compounds (inclusive of doxorubicin) showed a Log *P* varying from 0.567 to 4.137, rotatable bonds from 5 to 12, polar surface area from 195.1 A^2^ to 206.1 A^2^, and water solubility from 0.00873 mg/L to 390 mg/L. Analysis of similarity (ANOSIM), hierarchical cluster analysis, and neighbor-joining cluster analysis elucidated relationships among the drugs that are useful for pharmaceutical consideration. *Results and Discussion*. Although the new derivatives share the same parent scaffold (doxorubicin), elucidation by analysis of similarity (ANOSIM) indicates that these assorted compounds are substantially distinct. The number of oxygen and nitrogen atoms (hydrogen bond acceptors) remained constant at 12 for compounds. Although violations of the Rule of five remained constant at three for all compounds, the variation of Log *P* and water solubility offers potentially beneficial medicinal activity for this group of anticancer agents that may enhance the antitumor activity of these anthracycline antibiotics. Hierarchical cluster analysis results clearly differentiated the parent doxorubicin from all higher molecular weight analogs. This outcome is confirmed with the use of neighbor-joining cluster analysis. *Conclusion*. By utilizing in silico optimization with pattern recognition analysis, potentially advantageous analogs can be elucidated from known effective pharmaceuticals.

## 1. Introduction

 There are various types of breast cancer that will have different levels of proliferation, aggressiveness, and genetic constitution. The survival rate from breast cancer varies depending on these three factors. Treatment includes combinations of the following: surgery, drugs including hormone therapy, chemotherapy, and targeted therapies, and radiation therapy. Doxorubicin is an anthracycline antibiotic that does inhibit the proliferative growth of bacteria but is not applied in that clinical treatment due to the substantial level of toxicity to human physiology [1]. 

 The anthracycline antibiotic doxorubicin is utilized for the clinical treatment of other cancers such as hematological malignancies, various carcinomas, and soft tissue sarcomas [1–3]. The drug doxorubicin has a molecular structure that is light sensitive and usually administered intravenously [1–3]. Although the anthracyclines are some of the most effective anticancer agents, with effectiveness against more types of cancers than any other class of chemotherapeutic agents, they have a substantial problematic cardiotoxicity that increases as survival increases [4–6]. 

 Utilizing doxorubicin in combination with paclitaxel for the treatment of metastatic breast cancer produces beneficial outcome with over 90% response rate [7]. The problematic appearance of neutropenia and mucositis complicates treatment regimen and outcome results. Use of anthracycline drugs can incur congestive heart failure which can be identified in about 20% of the patients [7]. Appearance of mucositis and neutropenia can place dose limitations on the use of doxorubicin, even in combination with paclitaxel [8]. 

 Other studies have shown that the application of doxorubicin in liposomal form may reduce toxicity usually associated with anthracyclines [9]. The use of liposomal doxorubicin with a platinum agent may benefit therapy of advanced malignant epithelial ovarian carcinoma [9]. Encouraging results have been obtained for treatment of advanced solid tumors by pegylated liposomal doxorubicin with paclitaxel [10] having a reduced appearance of neutropenia and cardiotoxicity, with acceptable toxicity [10]. In other studies, the use of pegylated liposomal doxorubicin with platinum also demonstrated decreased neurotoxicity but encouraging outcomes for ovarian cancer patients [11]. 

 The investigation of novel designs of anthracycline antibiotics may benefit therapeutic approaches to the treatment of breast cancer. Presented here is the application of in silico optimization with pattern recognition analysis to improve pharmaceutical activity of already proven antineoplastic compounds.

## 2. Experimental Section

### 2.1. Molecular Modeling and Assembly of Constructs

 Numerical values of properties and molecular modeling was accomplished utilizing ACD/ChemSketch modeling v. 10.00 (Advanced Chemistry Development, 110 Yonge Street, Toronto, ON, Canada M5C 1T4). Other properties: polar surface area, violations of Rule of 5, molecular volume, number of oxygens, nitrogens, amines, hydroxyls, and so forth were determined using Molinspiration (Molinspiration Cheminformatics, Nova ulica 61, SK-900 26 Slovensky Grob, Slovak Republic). In silico structure search for substituent replacement was accomplished using Chemical substructure and similarity search with MolCart Chemical Data Base (Molsoft L. L. C., 3366 North Torrey Pines Court, Suite 300, La Jolla, Calif, USA). 

### 2.2. Pattern Recognition and Elucidation

 To identify underlying associations/patterns within the properties, numerical matrix required the use of various pattern recognition techniques. Included in the analysis is hierarchical cluster analysis accomplished by KyPlot v. 2.0 Beta 15 (Koichi Yoshioka 1997–2001). Neighbor-joining cluster analysis and ANOSIM (analysis of similarity) accomplished by PAST v. 1.80 (Oyvind Hammer, D. A. T. Harper 1999–2008). 

### 2.3. Numerical Analysis of Multivariate Data Matrix

Statistical analysis of all numerical data was performed by Microsoft EXCEL (EXCEL 2003, 1985–2003). Correlation analysis by Pearson *r* was done for some descriptors and was accomplished by GraphPad Software (GraphPad Instat v. 3.00 for Windows 95 GraphPad Software, San Diego, Calif, USA).

## 3. Results

**Figure 1:**
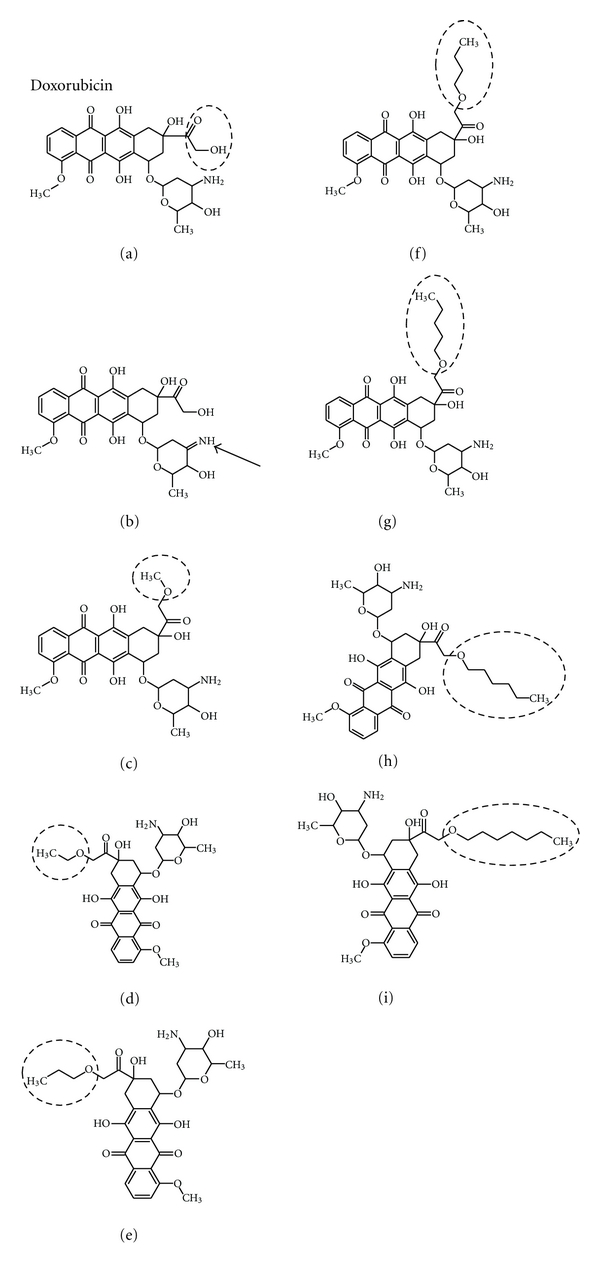
Comparative structures of doxorubicin and eight analogs. Note that doxorubicin (drug A) possesses a hydroxyl ketone group (see insert circle) that can be varied widely to form other compounds with variation in Log *P* and aqueous solubility. Drug B retains this hydroxy ketone group; however, an imine group replaces the original primary amine group on the daunosamine sugar substituent (see insert arrow). All other compounds form an alkoxy (–OR) homologous series of doxorubicin at the former hydroxy ketone group.

 The parent doxorubicin molecular structure as well as the derivatives formed from that scaffolding are shown in Figure 1. All compounds retain the flat planar chromophore region of this intercalating agent for insertion between two bases of DNA. Drug B design features an imine group (indicated by inset arrow) replacing the former primary amine group of the daunosamine sugar. Drugs C, D, E, F, G, H, and I are a homologous series of derivatives having an alkoxy group (–OR) replacing the former hydroxy ketone functional group of doxorubicin.

 Molecular descriptors for doxorubicin and the derivatives considered here were determined and presented in Table 1. The number of violations of the Rule of 5 remains at three; an expected outcome on account of variation occurs only on the daunosamine sugar (drug B) or the hydroxy ketone group. The number of rotatable bonds increases as the length of the aliphatic alkoxy substituent as do the formula weight, molecular volume, and Log *P* (becoming more lipophilic as the aliphatic alkoxy group extends in length). The number of oxygen and nitrogen atoms (hydrogen bond acceptors) remains constant at 12 throughout this assortment of compounds.

**Figure 2:**
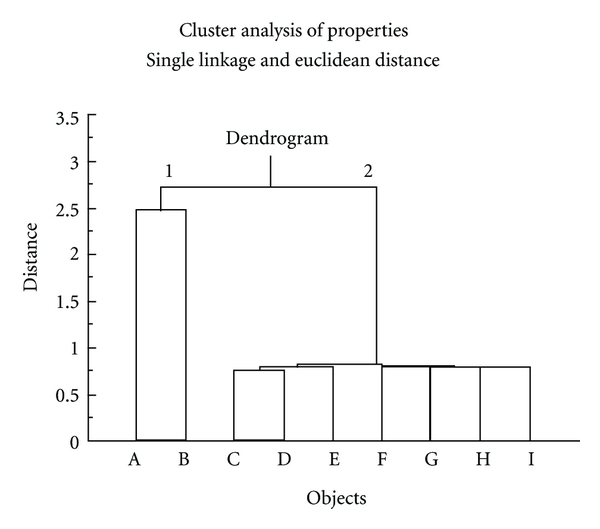
A dendrogram plot of hierarchical cluster analysis utilizing single linkage grouping method and Euclidean distance measurement. Note that this analysis clearly distinguishes drugs C, D, E, F, G, H, and I from the parent doxorubicin (drug A). Only drug B is determined to be highly similar to doxorubicin. Doxorubicin and drug B are join at supercluster 1, with remaining analogs associated to supercluster 2.

**Figure 3:**
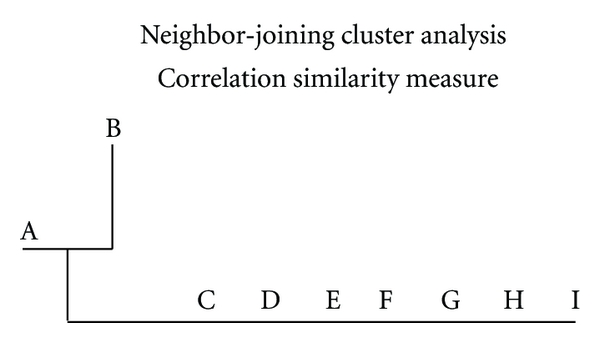
Results of neighbor-joining cluster analysis show drug A (doxorubicin) and the imine derivative drug B fall on the same node. Homologous series derivatives C, D, E, F, G, H, and I fall adjacently and essentially identically on a central node.

 Outcome of cluster analysis of compounds shown in Figure 1 is presented by 2-way dendrogram in Figure 2, which utilizes Euclidean distance measure (shortest distance) and single linkage clustering (the distance between two clusters is computed as the distance between the two closest elements in the two clusters). This analysis clearly shows that homologous series drugs C, D, E, F, G, H, and I are most similar to each other but distinct from doxorubicin. This outcome corroborated by neighbor-joining cluster analysis (Figure 3).

 Analysis of similarity (ANOSIM) indicates these assorted compounds are substantially distinct from doxorubicin.

## 4. Discussion

Alteration of substituents on the molecular structure of biologically active agents has been shown to have substantial effects on the pharmaceutical properties [12]. Substituent modification can change the medicinal characteristics either beneficially or destructively in terms of clinical efficacy. Substantial number of studies have been completed showing correlation with some structure modifications directly to important medicinal attributes such as bioavailability, lipid solubility, aqueous solubility, and so forth [12]. Other investigators have previously shown a particular relevance of Log *P*, formula weight, and hydrogen bonding activity to the effectiveness of druglikeness. Examination or screening of many potential drug candidates is possible by statistical comparison to already proven but related medicinal compounds. This approach of screening drug candidates improves the success rate for selection to drug trials and eventual full development. 

One very successful screening method is known as the Rule of 5, in which Log *P*, formula weight, and hydrogen bonding activity are taken to be some multiple of the numeric value of five. Specifically, the criteria impute that violation of two or more of the parameters would signal problems in drug bioavailability. These criteria include the following [12]: (1) a Log *P* value of less than 5; (2) a formula weight less than 500 grams/mole; (3) no more than 10 hydrogen bond acceptors (oxygen and nitrogen atoms); (4) no more than 4 hydrogen bond donors (–OH and –NH_n_). Doxorubicin is a molecule that intercalates the DNA molecule by way of the planar aromatic chromophore portion of the molecule and with the daunosamine sugar resting in a minor groove with accompanied interaction on the flanking base pairs [1–3]. To pursue a useful modification of doxorubicin will require maintaining the planar chromophore region that effectively rests between two adjacent base pairs of DNA and the sugar substituent that successfully interacts with adjacent base pairs restraining the molecule in position.

 Homologous series of compounds have a particular advantage of having predictable properties, and extensive studies have shown that as the numeric series increase the medicinal activity also increases to a maximum of six or seven carbon chain length (–(CH_2_)_5_CH_3_ or –(CH_2_)_6_CH_3_) [13]. Lengthening the carbon chain of the alkoxy group (–OR) produces substantial variations in the molecular properties Log *P*, formula weight, rotatable bonds, molecular volume, and water solubility.

 Molecular descriptors for doxorubicin and the derivatives were determined and are presented in Table 1. Number of violations of the Rule of 5 remains at three; an expected outcome on account of variation occurs only on the daunosamine sugar (of drug B) or the hydroxy ketone group. This is a known restriction for anthracyclines and explains part of the rational for intravenous administration of this agent. Violations of Rule of 5 do not rule out the clear effectiveness of doxorubicin. The polar surface area of all the homologous series compounds (drugs C, D, E, F, G, H, and I) remains constant at 195.1 Angstroms^2^, a value that does not facilitate intestinal absorption [12]. 

 Water solubility of drug B is substantially higher at 390 mg/Liter than that of doxorubicin itself at 92.84 mg/Liter. This is apparently due to the substitution of a primary amine group with an imine group that is covalently bonded to the daunosamine sugar (see Figure 1). As the length of the aliphatic alkoxy substituent increases, the water solubility decreases, which actually is an expected result caused by increased lipophilic tendency of the aliphatic branch. Correlation Pearson *r* for these descriptors reveals that Log *P* is directly correlated (*r* > 0.9500) to molecular weight, number of rotatable bonds, and molecular volume (coefficient of determination >0.9025, showing account of more than 90% of variance). Log *P* is inversely correlated to polar surface area, number of –OH and –NH_n_ (hydrogen bond donors), and water solubility. 

 Previous studies have demonstrated that values of Log *P* which are 2 ± 0.7 indicate the drug can penetrate the central nervous system [14]; this criteria include derivatives D, E, and F. A Log *P* value of 1.35 favors intestinal absorption, fulfilled by drug B. Colonic absorption is enabled by a Log *P* value of 1.32 [14], fulfilled by drug B. A Log *P* value between 3 and 4 promotes transdermal administration, fulfilled by drugs G and H. 

 Hierarchical cluster analysis is a pattern recognition method that sorts the subjects (drugs here) into groupings that follow criteria set by the investigator where the highly similar (by molecular properties of Table 1) drugs form distinct clusters [15]. Outcome of cluster analysis of drugs shown in Figure 1 is presented by dendrogram in Figure 2, utilizing Euclidean distance measure (shortest distance) and single linkage clustering (the distance between two clusters is computed as the distance between the two closest elements in the two clusters). This analysis clearly shows that homologous series drugs C, D, E, F, G, H, and I are most similar to each other but distinct from doxorubicin. Drug B is determined to be most similar to doxorubicin. This outcome suggests that drug B may behave similarly to doxorubicin; however, the homologous series (C, D, E, F, G, H, and I) are distinct and may differ in activity than doxorubicin, a desired goal for the design of new antineoplastic agents. 

 Neighbor-joining cluster analysis is a bottom up clustering method that is an alternative for hierarchical cluster analysis, that was initially introduced for phylogenetic analysis [16]. Analysis of Table 1 properties and using correlation similarity measure, the results again identified drug B as most similar to doxorubicin (drug A) (see Figure 3). The homologous series of compounds C, D, E, F, G, H, and I are alike and joined identically. 

 Although the derivatives share the same parent scaffold (doxorubicin), elucidation by analysis of similarity (ANOSIM) indicates that these assorted compounds are substantially distinct from doxorubicin. The ANOSIM outcome R is calculated to be 1.000 (Euclidean distance measure). This value asserts that all compounds studied here are distinct from the parent scaffolding of doxorubicin. Albeit this conclusion discernable by inspection of some descriptor numerical values, this algorithm confirms that drugs B, C, D, E, F, G, H, and I can be expected to express differentiation in pharmaceutical activity and medicinal action in the treatment of breast cancer.

 Previous studies have shown that substitutions on the sugar moiety (C-3′ position) will produce agents that express antitumor activity [17]. Other work has synthesized compounds similar to those presented in this study that have substituted various substituents in place of the hydroxyl group (2-hydroxy) of the –C(O)CH_2_OH terminus [18]. These structures include the following: 2-thiophene acetate, benzyl carbonate, 2-methyl sulfonyl ethyl carbonate, and butyrate, all of which showed antitumor activity when tested in vitro [18]. It follows that emplacement of alkoxy groups as shown in Figure 1 will provide further useful drugs which increases the number of pharmaceutical options in the clinical treatment of breast cancer. These derivatives of doxorubicin encompass pharmaceutical properties that diversify the bioactivity of anthracycline-like anticancer agents with the potential for favorable patient outcome. 

## 5. Conclusion

 In summation, eight derivatives of doxorubicin were elucidated from the parent molecular structure of doxorubicin. By developing derivatives of doxorubicin, the Log *P* values that result suggests an improved potential for drug B (imine derivative) to accomplish intestinal absorption and colonic absorption. A Log *P* value between 3 to 4 promotes transdermal administration, fulfilled by drugs G and H. For homologous series C, D, E, F, G, H, and I, the increasing length of the aliphatic alkoxy substituent increases the lipophilicity (thereby increasing Log *P*), increases molecular volume, and increases formula weight, but decreases water solubility. Violations of the Rule of 5 remain at three for all compounds. Modifications in the structure of doxorubicin vary the Log *P* property and potentially change the biological activity of these anthracycline antibiotics.

## Figures and Tables

**Table 1:** Molecular properties of derivatives.

Drug	Log *P*	Polar surface area (A^2^)	Molecular weight	Number of oxygens and nitrogens	Number of –OH and –NH	Violations of rule of five	Number of rotatable bonds	Molecular volume (A^3^)	Water solubility (mg/L)
Doxorubicin, A	0.567	206.1	543.5	12	7	3	5	459.2	92.8
B	1.39	203.9	541.5	12	6	3	5	453.4	390.0
C	1.18	195.1	557.5	12	6	3	6	476.8	10.2
D	1.55	195.1	571.6	12	6	3	7	493.5	3.15
E	2.06	195.1	585.6	12	6	3	8	510.3	0.971
F	2.62	195.1	599.6	12	6	3	9	527.1	0.299
G	3.12	195.1	613.7	12	6	3	10	543.9	0.0921
H	3.63	195.1	627.7	12	6	3	11	560.7	0.0283
I	4.13	195.1	641.7	12	6	3	12	577.5	0.00873

A^2^ is Angstroms^2^.

A^3^ is Angstroms^3^.
